# Nobiletin and Eriodictyol Suppress Release of IL-1β, CXCL8, IL-6, and MMP-9 from LPS, SARS-CoV-2 Spike Protein, and Ochratoxin A-Stimulated Human Microglia

**DOI:** 10.3390/ijms26020636

**Published:** 2025-01-14

**Authors:** Irene Tsilioni, Duraisamy Kempuraj, Theoharis C. Theoharides

**Affiliations:** 1 Laboratory of Molecular Immunopharmacology and Drug Discovery, Department of Immunology, Tufts University School of Medicine, Boston, MA 02111, USA; 2 Center of Excellence for Neuroinflammation Research, Institute for Neuro-Immune Medicine, Dr. Kiran C. Patel College of Osteopathic Medicine, Nova Southeastern University, Ft. Lauderdale, FL 33328, USA

**Keywords:** CXCL8, flavonoids, IL-1β, IL-6, long COVID, luteolin, mast cells, microglia, MMP-9, neuroinflammation, nobiletin, SARS-CoV-2 spike protein

## Abstract

Neuroinflammation is involved in various neurological and neurodegenerative disorders in which the activation of microglia is one of the key factors. In this study, we examined the anti-inflammatory effects of the flavonoids nobiletin (5,6,7,8,3′,4′-hexamethoxyflavone) and eriodictyol (3′,4′,5,7-tetraxydroxyflavanone) on human microglia cell line activation stimulated by either lipopolysaccharide (LPS), severe acute respiratory syndrome coronavirus 2 (SARS-CoV-2) full-length Spike protein (FL-Spike), or the mycotoxin ochratoxin A (OTA). Human microglia were preincubated with the flavonoids (10, 50, and 100 µM) for 2 h, following which, they were stimulated for 24 h. The inflammatory mediators interleukin-1 beta (IL-1β), chemokine (C-X-C motif) ligand 8 (CXCL8), IL-6, and matrix metalloproteinase-9 (MMP-9) were quantified in the cell culture supernatant by enzyme-linked immunosorbent assay (ELISA). Both nobiletin and eriodictyol significantly inhibited the LPS, FL-Spike, and OTA-stimulated release of IL-1β, CXCL8, IL-6, and MMP-9 at 50 and 100 µM, while, in most cases, nobiletin was also effective at 10 µM, with the most pronounced reductions at 100 µM. These findings suggest that both nobiletin and eriodictyol are potent inhibitors of the pathogen-stimulated microglial release of inflammatory mediators, highlighting their potential for therapeutic application in neuroinflammatory diseases, such as long COVID.

## 1. Introduction

Neuroinflammation, particularly pathogen-stimulated microglial activation, is a key pathological feature in numerous neurodegenerative diseases, including Alzheimer’s disease (AD) and Parkinson’s disease (PD) [1,2,3]. Microglia, the resident immune cells of the central nervous system (CNS), are essential for brain functions, especially the surveillance of the microenvironment and the maintenance of brain homeostasis, as well as defense against infections and injury [4]. When stimulated by pathogens, such as bacterial lipopolysaccharides (LPS), viral proteins, and other environmental toxins, microglia can initiate a cascade of responses aimed at protecting the brain, but prolonged activation can lead to the release of various inflammatory mediators, ultimately leading to neuronal damage and contributing to the development of chronic neuroinflammatory [5] and neurodegenerative disorders [6,7]. Infection with severe acute respiratory syndrome coronavirus 2 (SARS-CoV-2) causes the loss of microglial homeostasis [3]. The pro-inflammatory molecules evaluated in this study include the cytokines interleukin-1 beta (IL-1β) and IL-6, the chemokine (C-X-C motif) ligand 8 (CXCL8)/IL-8, and matrix metalloproteinase-9 (MMP-9), which disrupts neuronal connectivity [8,9,10,11]. Inflammatory molecules derived from microglia can impair the blood-brain barrier (BBB), allowing the entry of peripheral immune cells, activating and damaging glial cells and neurons, and contributing to neurodegenerative processes [12,13]. Perineural nets were reported to be phagocytosed by MMP-9-expressing microglia in the SOD1^G83A^ mouse strain, an Amyotrophic lateral sclerosis (ALS) disease model [14].

A high level of MMP-9 has been shown to exacerbate neuroinflammatory processes, neurodegeneration, basement membrane degradation, and BBB disruption [8,9,11,15,16]. Serum levels of MMP-9 levels were elevated in patients with coronavirus disease 2019 (COVID-19) [17,18] and associated with severe symptoms [19,20]. Blood MMP-9 levels were also reported to be higher in the acute phase of neuro COVID patients [16].

Mycotoxins have been increasingly reported to have neurotoxic effects [21,22]. Ochratoxin A (OTA), a mycotoxin found in mold in the environment and on foods, has immunotoxic and neurotoxic properties since it can cross the BBB and accumulate in brain areas, leading to neurodegeneration [22,23]. In a recent study, we found that OTA stimulated the release of IL-1β, IL-18, and CXCL8 from the human SV-40 microglia cell line [24].

Recent studies have highlighted the potential of natural compounds, especially citrus peel flavonoids [25,26], to modulate microglial activation and attenuate neuroinflammation [13,27,28,29]. We previously reported that luteolin (3′,4′,5,7-tetrahydroxyflavone, Lut) and methoxy luteolin (3′,4′,5,7-tetramethoxyflavone, Methlut) inhibited IL-1β release from cultured human SV-40 microglia stimulated by neurotensin [30] and MMP-9 release stimulated by the SARS-CoV-2 Spike protein [31]. Moreover, luteolin induced an anti-inflammatory and neuroprotective phenotype in BV-2 microglia [32]. Luteolin has been explored for the treatment of various conditions [33,34,35,36] including brain disorders [37,38,39,40,41], such as long COVID [42,43,44,45,46,47] and autism spectrum disorder (ASD) [48,49].

Nobiletin (5,6,7, 8,3′,4′-hexamethoxyflavone) found in citrus fruits has emerged as a promising candidate due to its anti-inflammatory, antioxidant, and neuroprotective properties and promotion of neuronal survival [50,51]. Eriodictyol (3′,4′,5,7-tetraxydroxyflavanone) also has antioxidant, anti-inflammatory, and neuroprotective properties [52]. However, the specific role of nobiletin or eriodictyol in pathogen-stimulated microglial-dependent inflammation remains poorly understood.

In this study, we investigated the potential of nobiletin and eriodictyol to inhibit the release of IL-1β, IL-6, CXCL8, and MMP-9 from the cultured human microglia cell line stimulated by pathogen-derived LPS, full-length Spike (FL Spike), and OTA.

## 2. Results

### 2.1. Inhibitory Effects of Nobiletin and Eriodictyol on LPS-Stimulated Microglia

**Figure 1:**
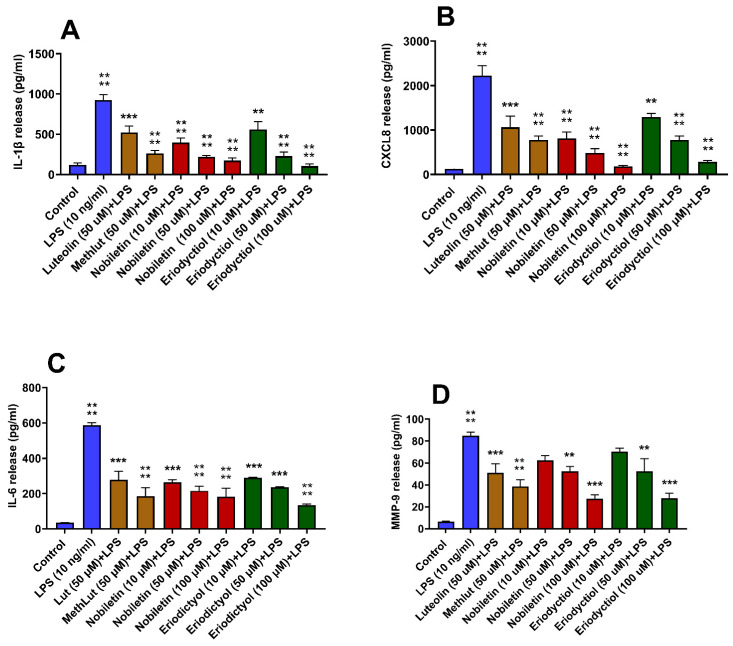
Inhibition of the release of inflammatory mediators from human microglia stimulated by LPS. Human microglia (0.5 × 10^5^ cells/1 mL/well in 24-well plates) were first preincubated with nobiletin (>98% purity; 10, 50, and 100 μM), eriodictyol (10, 50, and 100 μM), luteolin (50 μM), and methoxyluteolin (50 μM) for 2 h and then incubated with LPS (10 ng/mL) for 24 h. Control cells were treated with an equal volume of culture medium. Then, the cell culture supernatant fluids were collected and assayed for IL-1β (**A**), CXCL8 (**B**), IL-6 (**C**), and MMP-9 (**D**) by commercial ELISA kits. All assays were performed in triplicate. LPS was compared to the control; each of the other conditions was compared to LPS (n = 3, * *p* < 0.05, ** *p* < 0.01, *** *p* < 0.001, **** *p* < 0.0001).

Pre-treatment of microglia with either nobiletin or eriodictyol at 10, 50, and 100 µM for 2 h significantly inhibited LPS-induced pro-inflammatory mediator release, with 100 µM showing the highest inhibition (Figure 1A–D). Both nobiletin and eriodictyol inhibited release even at 10 µM, with the exception of MMP-9 release. Pretreatment with either luteolin or methoxy luteolin at 50 μM also significantly inhibited the release of all the mediators (Figure 1A–D). Multivariant analysis among all flavonoids at 50 µM showed no significant difference.

### 2.2. Inhibitory Effects of Nobiletin and Eriodictyol on FL Spike-Stimulated Microglia

**Figure 2:**
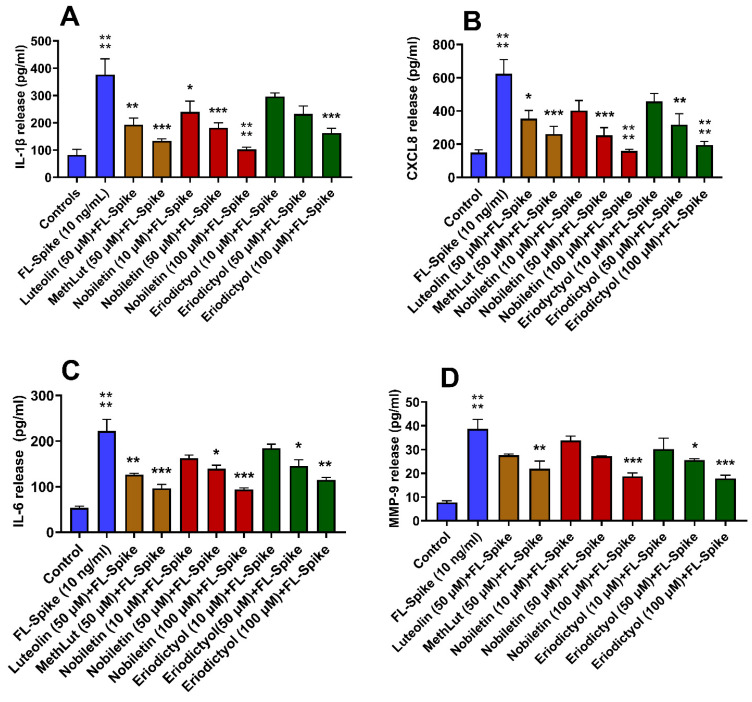
Inhibition of the release of inflammatory mediators from human microglia stimulated by SARS-CoV-2 Spike protein. Human microglia (0.5 × 10^5^ cells/1 mL /well in 24-well plates) were preincubated with nobiletin (10, 50, and 100 μM), eriodictyol (10, 50, and 100 μM), luteolin (50 μM), and methoxy luteolin (50 μM) for 2 h and then incubated with SARS-CoV-2 Spike protein (10 ng/mL) for 24 h. Control cells were treated with an equal volume of culture medium. Following this, cell culture supernatant fluids were collected and assayed for IL-1β (**A**), CXCL8 (**B**), IL-6 (**C**), and MMP-9 (**D**) by commercial ELISA kits. FL Spike was compared to the control; each of the other conditions was compared to FL Spike (n = 3, * *p* < 0.05, ** *p* < 0.01, *** *p* < 0.001, **** *p* < 0.0001).

Pre-treatment of microglia with either nobiletin or eriodictyol at 50 and 100 µM for 2 h significantly inhibited the FL Spike-induced release of IL-1β, CXCL8, IL-6, and MMP-9 (Figure 2A–D). Nobiletin at 10 µM inhibited LPS induced IL-1β release but not eriodictyol. Pre-treatment with either luteolin or methoxy luteolin at 50 μM also significantly inhibited the release of all the mediators (Figure 2A–D). Multivariant analysis among all flavonoids at 50 µM showed no significant difference.

### 2.3. Inhibitory Effects of Nobiletin and Eriodyctiol on OTA-Stimulated Microglia

**Figure 3:**
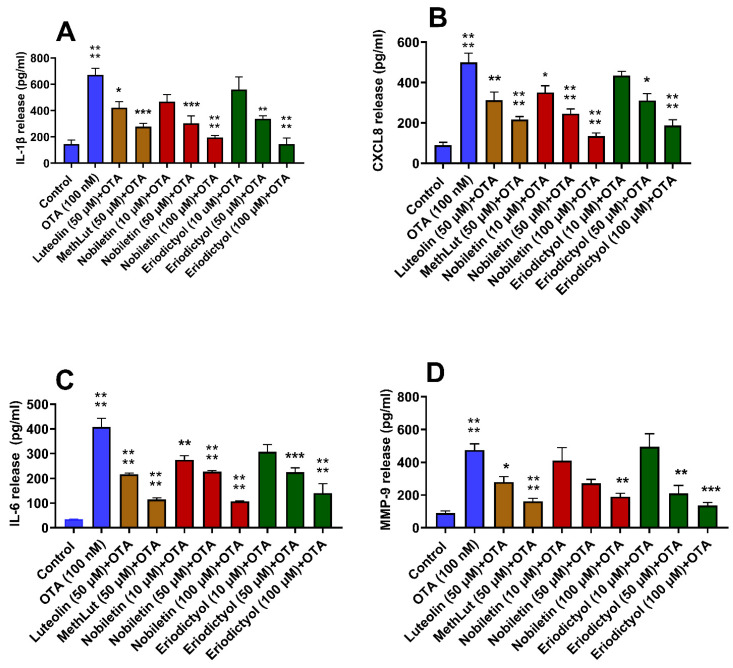
Inhibition of the release of inflammatory mediators from human microglia stimulated by OTA. Human microglia (0.5 × 10^5^ cells/500 μL/well in 24-well plates) were preincubated with nobiletin (10, 50, and 100 μM), luteolin (50 μM), and methoxy luteolin (50 μM) for 2 h and then incubated with OTA (10 nM) for 24 h (n = 3). Control cells were treated with an equal volume of culture medium. Following this, cell culture supernatant fluids were collected and assayed for IL-1β (**A**), CXCL8 (**B**), IL-6 (**C**), and MMP-9 (**D**) by commercial ELISA kits. OTA was compared to the control; each of the other conditions was compared to OTA (n = 3, * *p* < 0.05, ** *p* < 0.01, *** *p* < 0.001, **** *p* < 0.0001).

Pre-treatment of microglia with either nobiletin or eriodictyol at 50 and 100 µM for 2 h significantly inhibited the OTA-induced release of IL-1β, CXCL8, IL-6, and MMP-9 from microglia, with the 100 µM concentration showing the greatest inhibition (Figure 3A–D). Nobiletin, unlike eriodictyol, significantly inhibited the release of CXCL8 and IL-6, even at 10 µM, while neither inhibited the release of MMP-9 at this low concentration. Pretreatment with either luteolin or methoxy luteolin at 50 μM significantly inhibited the release of all the mediators (Figure 3A–D). Multivariant analysis among all flavonoids at 50 µM showed no significant difference.

## 3. Methods

### 3.1. Culture of Human Microglia

The immortalized human microglia SV-40 cell line (cat no. T10251), derived from primary human microglia, was obtained from Applied Biological Materials Inc. (ABM Inc., Richmond, BC, Canada) and maintained in Prigrow III medium (ABM Inc., Richmond, BC, Canada) supplemented with 10% fetal bovine serum (FBS) and 1% penicillin/streptomycin at 37 °C in a 5% CO_2_ incubator, as we have reported previously and recommended by the supplier [24]. Cells were cultured in type I collagen-coated T25 flasks (BD PureCoat™ ECM Mimetic Cultureware, Becton Dickinson, Bedford, MA, USA). The microglia-SV40 cell line retained its phenotype and proliferative capacity for over 10 passages. All the experiments were conducted using multiple cell thawings and subcultures that did not exceed 10 passages. Cell viability was assessed via the trypan blue (0.4%) exclusion method, and all experiments were performed in type I collagen-coated tissue culture plates (BD) with cultures over 95% viability.

### 3.2. Treatments of Microglia

Human microglia cell line (0.5 × 10^5^ cells in 1 mL complete medium/well in 24-well culture plate) were incubated for 24 h with the following stimuli: lipopolysaccharide (LPS; 10 ng/mL) (Sigma-Aldrich, St. Paul, MN, USA), full-length SARS-CoV-2 Spike protein (10 ng/mL), and OTA (10 ng/mL) (both from Abcam, Waltham, MA, USA). Luteolin, tetramethoxyluteolin (Methlut), hexamethoxyflavone (nobiletin), and tetrahydroxyflavanone (Eriodictyol), all >98% purity, were obtained from (CAS BioSciences, Costa Mesa, CA, USA). Levels of inflammatory mediators IL-1β, CXCL8, IL-6, and MMP-9 were quantified in the cell culture supernatant by enzyme-linked immunosorbent assay (ELISA) using commercial kits (BioTechne/R&D System, Minneapolis, MN, USA), following the manufacturer’s protocols, as we have reported previously [53,54]. Control cells were treated with an equivalent volume of culture medium in all experimental conditions. To investigate the inhibition of microglia activation, cells were pretreated with the various flavonoids at 10, 50, and 100 μM for 2 h.

### 3.3. Statistical Analysis

All experimental conditions were conducted in triplicate, and each experiment was repeated a minimum of three times (n = 3). Data are expressed as the mean ± standard deviation (SD). Multiple comparisons were performed using one-way analysis of variance (ANOVA) followed by Tukey’s multiple comparisons test. All statistical analyses were conducted using GraphPad Prism software version 10.0.3. A *p*-value of <0.05 was considered statistically significant in all comparisons.

## 4. Discussion

The present study demonstrates that both nobiletin (5,6,7,8,3′,4′-hexamethoxyflavone) and eriodictyol (3′,4′,5,7-tetraxydroxyflavanone) inhibited the LPS-, SARS-CoV-2-, or OTA-induced release of IL-1β, CXCL8, IL-6, and MMP-9 from human microglia stimulated by three different pathogenic agents: LPS, FL Spike, and OTA. Previous studies have reported that nobiletin inhibited the release of proinflammatory cytokines from LPS-stimulated mouse microglia [55,56]. Eriodictyol has also been shown to suppress LPS-stimulated BV-2 microglia [57]. There was no significant difference between nobiletin and eriodictyol at 100 μM. Multivariant analysis at 50 μM did not show any significance compared to luteolin (3′,4′,5,7-tetrahydroxyflavone) and methoxyluteolin (3′,4′,5,7-tetramethoxyflavone), which were used as “positive controls” because we previously reported them to inhibit IL-1β release from cultured human SV-40 microglia stimulated by neurotensin [30] and MMP-9 release stimulated by the FL Spike protein [31].

We previously reported that the SARS-CoV-2 Spike protein stimulated cultured human microglia SV-40 to secrete IL-1β, IL-18, MMP-9, and protein S100B, all of which are linked to brain damage [58]. Additional evidence indicates that the Spike protein can directly activate microglia [59,60,61], leading to proinflammatory effects. We also showed that SV-40 microglia release MMP-9 when stimulated by the Spike protein [58]. The Spike protein was also shown to induce neuroinflammation in mice via the activation of NLPR3 (nucleotide-binding oligomerization domain, Leucine-rich Repeat, and Pyrin domain-containing) and the BV-2 murine microglia cell line in vitro [62].

We recently reported that OTA stimulated the release of IL-1β, IL-18, and CXCL8 from the human SV-40 microglia cell line [24].

The present findings are supported by other previous studies showing that polyphenolic compounds can lower MMP-9 levels in both in vivo and in vitro conditions [63,64,65]. One recent study reported that methoxylated flavones inhibited the tumor necrosis factor (TNF)-mediated induction of MMP-9 [15].

The flavonoids examined in this study have been considered for therapeutic intervention for a number of neurodegenerative conditions [66]. For instance, nobiletin could serve as a potential inhibitor of beta amyloid (Aβ) toxicity [67]. In particular, nobiletin prevented Aβ 1-40 peptide-induced neuroinflammation and cognitive decline [68] and the Aβ 25-35 peptide-induced death of cultured primary neurons in vitro [69]. Nobiletin may also be beneficial in AD [70,71] by reducing neuroinflammation [56,72,73]. Nobiletin has also been shown to be useful in PD [72,73,74,75]. Moreover, nobiletin induced neurite outgrowth in PC12D cells, a rat pheochromocytoma cell line [76]. Eriodictyol was reported to reduce neuroinflammation induced by experimental stroke in rodents [77]. Eriodictyol also improved memory in Aβ 25-35-treated mice [78].

The mechanism of inhibition by the flavonoids is not entirely clear. Nobiletin was reported to inhibit mitogen-activated protein kinases (MAPK) and nuclear factor-kappa B (NF-kB) signaling pathways [55], as previously been reported for Methlut [79]. Both nobiletin [80,81] and eriodictyol [78,82] were reported to inhibit the NLP3 inflammasome, as did luteolin [83]. Such flavonoids could also inhibit activation of the cellular regulatory complex mammalian target of rapamycin (mTOR), as shown for for nobiletin [84] and Methlut [30,85,86]. 

Flavonoids are not soluble in aqueous media, making their administration and oral absorption in sufficient amounts problematic. An advantage of eriodictyol over other flavonoids is its planar configuration, which renders it partially water-soluble and, hence, easier to formulate in effective concentrations for delivery where other solvents may be irritating.

## 5. Conclusions

These findings indicate that both nobiletin and eriodictyol are potent inhibitors of the pathogen-stimulated microglial release of inflammatory mediators, highlighting their potential for therapeutic application in neuroinflammatory disorders.

## Data Availability

Data will be provided upon reasonable request.
